# Predicting In-Hospital Antibiotic Use in the Medical Department: Derivation and Validation Study

**DOI:** 10.3390/antibiotics11060813

**Published:** 2022-06-16

**Authors:** Itamar Poran, Michal Elbaz, Adi Turjeman, Maayan Huberman Samuel, Noa Eliakim-Raz, Jeries Nashashibi, Mical Paul, Leonard Leibovici

**Affiliations:** 1 Department of Medicine E, Rabin Medical Center, Beilinson Hospital, Petach Tikva 4941492, Israel; michalelbaz12@gmail.com (M.E.); aditur88@gmail.com (A.T.); maayan.hu@gmail.com (M.H.S.); noaeliakim@gmail.com (N.E.-R.); leibovici@clalit.org.il (L.L.); 2 Sackler Faculty of Medicine, Tel Aviv University, Tel Aviv 6997801, Israel; 3 Infectious Diseases Unit, Rabin Medical Center, Beilinson Hospital, Petah Tikva 4941492, Israel; 4 Department of Medicine D, Rambam Health Care Campus, Haifa 3436212, Israel; jeryes@gmail.com; 5 Infectious Diseases Institute, Rambam Health Care Campus, Haifa 3436212, Israel; m_paul@rambam.health.gov.il; 6 The Ruth and Bruce Rappaport Faculty of Medicine, Technion-Israel Institute of Technology, Haifa 3436212, Israel

**Keywords:** antibiotic prescription, antibiotic stewardship, epidemiology, prediction model

## Abstract

Background: The rise of multi-drug-resistant pathogens and nosocomial infections among hospitalized patients is partially attributed to the increased use of antibiotic therapy. A prediction model for in-hospital antibiotic treatment could be valuable to target preventive strategies. Methods: This was a retrospective cohort study, including patients admitted in 2018 to medical departments and not treated with antibiotics during the first 48 h. Data available at hospital admission were used to develop a logistic model to predict the probability of antibiotic treatment during hospitalization. The performance of the model was evaluated in two independent validation cohorts. Results: In the derivation cohort, antibiotic treatment was initiated in 454 (8.1%) out of 5592 included patients. Male gender, lower functional capacity, prophylactic antibiotic treatment, medical history of atrial fibrillation, peripheral vascular disease, solid organ transplantation, chronic use of a central venous catheter, urinary catheter and nasogastric tube, albumin level, mental status and vital signs at presentation were identified as predictors for antibiotic use during hospitalization and were included in the prediction model. The area under the ROC curve (AUROC) was 0.72 (95% CI 0.70–0.75). In the highest probability group, the percentage of antibiotic treatment was 18.2% (238/1,307). In the validation cohorts, the AUROC was 0.73 (95% CI 0.68–0.77) and 0.75 (95% CI 0.72–0.78). In the highest probability group, the percentage of antibiotic treatment was 12.5% (66/526) and 20.7% (244/1179) of patients. Conclusions: Our prediction model performed well in the validation cohorts and was able to identify a subgroup of patients at high risk for antibiotic treatment.

## 1. Introduction

Sepsis is one of the leading causes of in-hospital death, affecting approximately 1.7 million adults in the United States, with up to 33% of in-hospital deaths being associated with sepsis [1,2,3]. A cornerstone in the fight against sepsis is its rapid recognition and the initiation of empirical antibiotic treatment [4]. As a result, during the past few decades, the threshold for applying antibiotic treatment has dropped and antibiotic use has been on the rise, in particular, broad-spectrum antibiotics, such as third-generation cephalosporins and carbapenems [5,6].

Despite its importance, improper use of antibiotics may result in devastating results. Excessive use of antibiotics is a major risk factor for the development of multi-drug-resistant pathogens, a global danger that increases over the years, leaving physicians with limited resources for treatment and costing the lives of many patients. A study assessing the prevalence of antibiotic use in 10 acute care hospitals in the United States estimated that up to 50% of hospitalized patients will be treated with at least one type of antibiotic during hospitalization, of which up to 30% of inpatient days of antibiotic therapy is considered unnecessary [7]. A recent study evaluating the incidence of antibiotic-associated adverse drug events (ADEs) in hospitalized patients found that up to 20% of patients receiving systemic antibiotic therapy experienced at least one antibiotic-associated ADE [8]. Another concern is the risk of infections due to *Clostridoides difficile* (CD), a well-known nosocomial pathogen with a predilection to hospitalized patients under antibiotic treatment [9]. A study evaluating the time interval of increased risk for CD infection after exposure to antibiotic therapy found that during antibiotic therapy and in the first month after cessation of the therapy, patients had a 7–10-fold increased risk for infection [10].

In 2015, the World Health Organization recognized antibiotic resistance as a global health threat and published recommendations to reduce the excessive use of antibiotic treatment in health care facilities by employing a global surveillance system, prescription supervision, and antibiotic stewardship programs [11]. Variation in antibiotic use among and within different settings may reflect inappropriate use and was found to correlate with the prevalence of certain antibiotic-resistant bacteria [12]. Previous studies aiming to analyze in-hospital antibiotic use were ecological studies focused on hospital characteristics rather than the individual patient receiving the treatment [1,13,14,15].

In this study, we aimed to develop and validate a prediction model for in-hospital administration of antibiotic treatment in the medical department. We included patients who were hospitalized for at least 48 h, in which no antibiotic treatment was initiated during this period, to identify in advance the group of patients who will most benefit from antibiotic stewardship efforts before antibiotics are prescribed. Such a model would be of interest when looking for patients at risk for CD infection, or candidates for the removal of an unjustified label of allergy to an antibiotic. In addition, it can serve as an aid in prospective studies and randomized controlled trials of antibiotics. Finally, the model can be used in benchmarking efforts to detect unexplained variations in antibiotic prescription.

## 2. Material and Methods

### 2.1. Study Design, Participants, and Data Collection

We performed an observational, retrospective, cohort study of medical patients hospitalized in 2018. For the derivation cohort, we included all adult patients admitted from the emergency department to the medical departments of Beilinson Hospital (Beilinson). Beilinson is located in the center of Israel, a university hospital with 337 beds in its medical departments. We excluded elective hospitalization, patients hospitalized for less than 48 h, patients treated with antibiotics during their first 48 h of hospitalization, and patients admitted with the diagnosis of endocarditis, osteomyelitis, or bloodstream infection. For patients with re-hospitalizations during the study year, we included their first hospitalization. Patient flow diagram is available in the Supplementary Material. Data for the derivation cohort were extracted from the electronic patient record (EPR) and included demographic factors, medical history, chronic medical treatment, and clinical and laboratory parameters available at hospital admission.

### 2.2. Outcome

The primary outcome was antibiotic treatment first prescribed after at least 48 h from admission to the medical department. Antibiotic treatment was defined as at least one dose of antibiotic treatment given in an oral, parenteral, or intramuscular route. We excluded the use of antibiotics for purpose of prophylaxis including Trimethoprim-Sulfamethoxazole for *Pneumocystis pneumonia* prophylaxis, use of macrolides for respiratory infections prophylaxis, and periprocedural prophylaxis use of antibiotics.

### 2.3. Candidate Predictor Variables

We inspected candidate predictor variables including age, gender, functional status before hospitalization according to Katz index of independence in Activities of Daily Living (ADL) [16], medical history including diabetes, dyslipidemia, hypertension, ischemic heart disease, cerebrovascular event, malignancy, peripheral vascular disease, atrial fibrillation, congestive heart failure, chronic lung disease, chronic liver disease, chronic kidney disease, pressure wounds, immunosuppression state, chronic medication, and prophylactic antibiotic treatment before hospitalization. We also evaluated the chronic use of a nasogastric tube (NGT), a central venous catheter (CVC), an indwelling urinary catheter, and a history of any surgical procedure in the past 30 days before admission. Clinical and laboratory variables that are routinely measured and available in a triage setting were evaluated including mental status at presentation as assessed by the medical staff, heart rate, temperature, blood pressure, and oxygen saturation in room air. Laboratory variables at presentation to the hospital included blood count, liver and renal function tests, urine analysis, and blood gasses.

### 2.4. Model Development and Statistical Analysis

We did not perform a formal analysis of sample size. From preliminary investigation, we expected to include 5000 patients of which around 10% of patients were treated with antibiotics during hospitalization, numbers that seem sufficient to build a rich model that will include the explanatory variables without overfitting. The distribution of the continuous variables was assessed visually and by using the Kolmogorov–Smirnov normality test. As most continuous variables did not have a normal distribution, we present their values as median and their 25–75% percentiles. Mann–Whitney test was used to compare continuous variables. A Chi-square test was used for categorical variables.

A prediction model was established by using multivariate logistic regression analysis. All variables known within 24 h of hospital admission and associated with antibiotic administration on univariate analysis with statistical significance (*p* ≤ 0.1) and variables that make clinical sense were candidates for regression analyses and were entered with the backward elimination method. Before entering candidates into the regression logistic model, we examined multicollinearity using variation inflation factors, with a result of ≥2.5 considered high. Missing values were not imputed, and observations with missing values were excluded from the final analysis. After examining several possible models, the model with the lowest Akaike information criterion (AIC) was selected. The result of the logistic equation assigns each patient a probability (P) to be treated with antibiotics. Receiver operating characteristic (ROC) analysis together with its 95% confidence intervals (CI) was performed on these scores to assess the ability and the optimal cutoff value for discriminating between patients who received antibiotic treatment during their hospitalization and patients who did not. We used the probability quartile cutoff points and the Youden index to form four thresholds to predict a positive outcome and calculated the sensitivity, specificity, positive predictive value (PPV), negative predictive value (NPV), and accuracy for each cutoff point. All data were analyzed by using SPSS version 27. We report our results in concordance with the TRIPOD guideline [17].

### 2.5. Test of Model Performance and External Validation

To assess the model performance and its ability to discriminate the primary outcome of antibiotic treatment during hospitalization we externally validated the final prediction model in two external cohorts: all patients admitted to the medical departments in Hasharon Hospital (Hasharon) and Rambam Health Care Campus (Rambam) in 2018. Hasharon, located in the center of Israel in the city of Petah Tikva, is a community hospital with 114 beds in its medical departments. Hasharon uses the same EPR system as Beilinson. Rambam, located in the north of Israel in the city Haifa, is a university hospital with 350 beds in its medical departments. Rambam uses a different EPR than Beilinson and Hasharon. The same inclusion and exclusion criteria were applied to the validation cohorts. Patient flow diagram is available in the Supplementary Material. Discriminant analyses were performed using the ROC curve. The area under the curve (AUC) and its 95% confidence interval and the performance metrics for each cutoff point were calculated. Ethical approval was given by the hospital Ethics Committee in each center that took part in this study.

## 3. Results

### 3.1. Derivation Cohort—Beilinson Hospital

**Figure 1:**
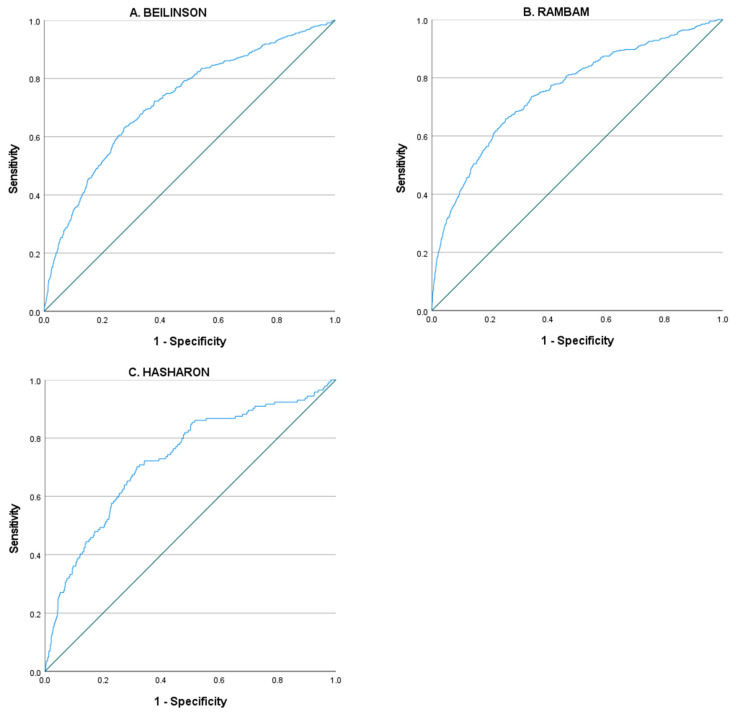
ROC curve of P (probability of antibiotic treatment as calculated by the logistic model shown in Table 2), (**A**) Derivation cohort Beilinson (area under the ROC curve 0.72 (95% CI 0.70–0.75)); (**B**) Validation cohort Rambam (area under the ROC curve 0.75 (95% CI 0.72–0.78)); (**C**) Validation cohort Hasharon (area under the ROC curve 0.73 (95% CI 0.68–0.77)).

In 2018, there were 12,656 admissions to the medical departments in Beilinson. After excluding repeated hospitalizations, patients treated with antibiotics during their first 48 h, and patients admitted with the diagnosis of osteomyelitis, endocarditis, or bloodstream infection, 5592 (44.1%) patient-unique hospitalizations were found eligible and were included in the derivation cohort. The median age was 71 years (interquartile range 60–80) and there were 2660 (47.6%) women. Patients’ demographics and baseline characteristics are summarized in Table 1. Out of the 5592 patients included in the derivation cohort, 454 (8.1%) patients were treated with antibiotics starting 48 h from admission to the hospital. The final logistic model is described in Table 2. The area under the ROC curve for the derivation cohort was 0.72 (95% CI 0.70–0.75), Figure 1. We used P quartiles cutoff points to divide the patients into four groups with ascending probability for antibiotic treatment: 50/1540 (3.2%); 58/1390 (4.2%); 108/1355 (8.0%); and 238/1307 (18.2%). The cutoff points for the P-value and numbers and percentages of treated patients are shown in Table 3. The performance metrics for each cutoff point are available in the Supplementary Material.

### 3.2. Validation Cohort—Hasharon Hospital

In Hasharon, in 2018, there were 4946 hospitalizations in the medical departments, out of which 3061 (61.8%) patient-unique hospitalizations were found eligible and were included in the validation cohort. The median age was 73 years (interquartile range 61–83) and 1543 (50.4%) were women. Patients’ characteristics and variables of the predictive model are shown in Table 4. Out of the 3061 patients included, 144 (4.7%) were treated with antibiotics starting 48 h from presentation to the hospital. We used the logistic equation to calculate the probability for the outcome of antibiotic treatment during hospitalization (P). In this population, the area under the ROC curve was 0.73 (95% CI 0.68–0.77)—Figure 1. Using the cutoffs for the probability that were derived from the derivation cohort, the percentages of antibiotic treatment in the four groups were: 19/1115 (1.7%); 21/830 (2.5%); 38/590 (6.4%); and 66/526 (12.5%)—Table 3. The performance metrics for each cutoff point are available in the Supplementary Material.

### 3.3. Validation Cohort—Rambam Health Care Campus

In Rambam, in 2018, there were 10,734 hospitalizations in the medical departments, out of which 4494 (41.8%) patient-unique hospitalizations were found eligible and were included in the validation cohort. The median age was 67 years (interquartile range 53–79) and 2050 (45.6%) were women. Patients’ characteristics and variables of the predictive model are shown in Table 4. Out of the 4494 patients included, 390 (8.7%) were treated with antibiotics starting 48 h from hospital admission. In this population, the area under the ROC curve was 0.75 (95% CI 0.72–0.78)—Figure 1. Using the cutoffs for the probability that were derived from the derivation cohort, the percentages of antibiotic treatment in the four groups were: 14/549 (2.6%); 48/1392 (3.4%); 84/1374 (6.1%); and 244/1179 (20.7%)—Table 3. The performance metrics for each cutoff point are available in the Supplementary Material.

## 4. Discussion

In this study, we developed a prediction model for antibiotic treatment in the medical department initiated after 48 h of hospital stay. The discrimination performance of the model was good with an AUC of 0.72 (95% CI 0.70–0.75). In addition, the model was externally validated and found to be stable in two independent cohorts. We were able to define a high-risk subgroup of patients with a probability for the outcome range between 12.5% and 20.7%.

In our study, we tried to limit the inclusion for patients without a clear and binding reason for antibiotic treatment. During the examination of the explanatory variables for the model, we attempted to identify those who are universal, easily available on presentation to the hospital and whose presence does not compel the physician to initiate antibiotic treatment so the model can be activated in the first 48 h of admission.

We aimed to identify a subgroup of patients who would most likely benefit from an antibiotic stewardship program. We chose to present four subgroups with an increased probability of antibiotic treatment to emphasize the connection between the explanatory risk factors in the model and antibiotic treatment in the high-risk group of patients. We believe that this group of patients can gain from such intervention, even if antibiotic treatment was not prescribed.

Some of the variables included in the model are characteristics of frailty: reduced functional capacity, impaired mental status, and medical history of cardiovascular disease, including atrial fibrillation and peripheral vascular disease, which are features of this vulnerable population, known to be at increased risk for in-hospital adverse outcomes and iatrogenic complications [18].

Other variables included in the model are well-known risk factors for the adverse outcomes of antibiotic treatment: prior antibiotic treatment, nasogastric tube, and low albumin level are all risk factors for *Clostridoides difficile* infection [19,20]. Peripheral vascular disease, central venous catheter, indwelling urinary catheter, and prior antibiotic treatment are risk factors for multidrug-resistance infection [21,22,23].

Our study has limitations. We used data collected retrospectively on hospitalized patients in 2018, and variations that occurred during this year in terms of morbidity and epidemic could impair the model’s ability to predict in others. To our knowledge, no such variations took place in Israel during that time.

We only used data collected retrospectively from EPR. We tried to include explanatory variables that are easily available at presentation for clinical decision making considering that the model will be implemented in the electronic record system. Data and variables that were not available for extraction from EPR were not included in this analysis. Other EPRs might not include some of the variables used in our model.

Although we tried to include uniform and explicit variables, some of the included ones are subject to clinical judgment. Information regarding the patient’s mental status and functional capacity was retrieved according to the evaluation of the medical staff and may not be consistent in different hospitals. Nevertheless, as we assessed full mental status and full functional capacity vs. all others, we believe these variables may still quickly and easily be used in clinical practice.

Bearing in mind that the type of patients and the indications for treatment in the different departments vary from one another, we only included patients admitted from the emergency department to the medical department. As a result, the model may not apply to patients from other departments or elective patients. It is important to note that in Israel, the emergency department is the most common source of medical admissions.

In our derivation cohort, we included patients from Beilinson Hospital, the largest organ transplant center in Israel. As such, Beilinson medical wards encompass many transplant patients with their unique complications that may diverge from patients in other hospitals. In the derivation cohort, solid organ transplantation was found as a significant predictor for the outcome with OR 1.82 (95% CI 1.11–2.98). Despite the difference in this and other characteristics among the patients in the included centers, the external validation of the model and its good performance in two independent, community, and tertiary-level hospitals gives us high confidence in its predictive ability in other centers.

We intended to build a prediction model that can identify the patients with the highest risk for antibiotic treatment and can be easily incorporated into the electronic medical system of patients in different hospitals. This type of model can be of value for clinical decision making and can be used as an “alert” for infection control as part of the local antibiotic stewardship program. In addition, this model can be used to compare antibiotic prescriptions at different sites and can be used for epidemiological research in times or sites with increased incidence of nosocomial infections. Finally, this type of model, along with its explanatory variables, emphasizes once more the importance of the known risk factors for adverse outcomes, which hold the improper use of antibiotic treatment.

In conclusion, our prediction model performed well in the validation cohorts and was able to identify a sub-group of patients at high risk for antibiotic treatment.

## Figures and Tables

**Table 1:** Derivation cohort (Beilinson): demographics and underlying disorders of patients with and without antibiotic treatment during hospitalization.

Characteristic	Antibiotic Treatment (NO) N = 5138	Antibiotic Treatment (YES) N = 454	*p*-Value
Gender, female	2461 (47.9%)	199 (43.8%)	0.096
Age, years	71 (60–80)	73 (64–82)	<0.001
Hospitalization, days	3 (2–6)	12 (8–20)	<0.001
Amount of chronic medication	6 (3–9)	7 (4–9)	0.001
Prophylactic antibiotic treatment	181 (3.5%)	35 (7.7%)	<0.001
Body temp (°C)	36.7 (36.5–36.9)	36.8 (36.6–37.1)	<0.001
Heart rate	80 (69–94)	85 (74–96)	<0.001
Systolic blood pressure (mmHg)	136 (120–152)	133 (113–153)	0.003
Diastolic blood pressure (mmHg)	73 (62–83)	70 (59–81)	<0.001
O2 saturation (%)	98 (97–100)	98 (96–100)	<0.001
White blood cell count (cells/µL)	8.15 (6.44–10.38)	8.87 (6.61–11.98)	<0.001
Hemoglobin (mg/dL)	12.6 (10.9–14.0)	11.9 (10.0–13.3)	<0.001
Platelet count (cells/µL)	236 (186–301)	244 (181–309)	0.669
Albumin (mg/dL)	4.2 (3.8–4.5)	3.8 (3.4–4.2)	<0.001
Neutrophils (cells/µL)	5.6 (4.2–7.6)	6.5 (4.3–9.3)	<0.001
Creatinine (mg/dL)	0.95 (0.76–1.32)	1.04 (0.76–1.57)	0.012
Urea (mg/dL)	40 (29–59)	47 (33–78)	<0.001
Full functional capacity	3063 (59.6%)	163 (35.9%)	<0.001
Full mental status	4660 (90.7%)	339 (74.7%)	<0.001
Steroidal treatment at admission	474 (9.2%)	58 (12.8%)	0.013
Solid-organ transplantation	156 (3.0%)	22 (4.8%)	0.035
Diabetes mellitus	1521 (29.6%)	148 (32.6%)	0.181
Insulin treatment	668 (13%)	72 (15.9%)	0.085
Chemotherapy 6 months before hospitalization	319 (6.2%)	45 (9.9%)	0.002
Hypertension	2435 (47.4%)	231 (50.9%)	0.154
Ischemic heart disease	1115 (21.7%)	100 (22.0%)	0.872
Congestive heart failure	788 (15.3%)	77 (17.0%)	0.359
Chronic obstructive lung disease	299 (5.8%)	33 (7.3%)	0.21
Peripheral vascular disease	185 (3.6%)	29 (6.4%)	0.003
Cerebrovascular disease	931 (18.1%)	87 (19.2%)	0.581
Atrial fibrillation	787 (15.3%)	94 (20.7%)	0.003
Bronchiectasis	54 (1.1%)	11 (2.4%)	0.009
Diverticulosis	75 (1.5%)	5 (1.1%)	0.538
Liver cirrhosis	94 (1.8%)	18 (4.0%)	0.002
End-stage renal disease	105 (2.0%)	14 (3.1%)	0.141
Nasogastric tube	97 (1.9%)	46 (10.1%)	<0.001
Surgery 30 days before hospitalization	109 (2.1%)	17 (3.7%)	0.026
Pressure wounds	305 (5.9%)	73 (16.1%)	<0.001
Central venous catheter	78 (1.5%)	19 (4.2%)	<0.001
Urinary catheter	476 (9.3%)	112 (24.7%)	<0.001

Continuous variables are described as median (25–75 percentiles); discrete variables are described as number (%).

**Table 2:** Logistic model coefficients for calculating the probability of antibiotic treatment during hospitalization.

Variable	B	Odds–Ratio	95% CI	*p*-Value
Constant	1.777			
Gender, female	−0.293	0.746	0.608–0.916	0.005
Prophylactic antibiotic treatment	0.627	1.872	1.248–2.809	0.002
Heart rate *	0.006	1.006	1.001–1.011	0.014
Diastolic blood pressure (mmHg) *	−0.006	0.994	0.987–1.001	0.091
O2 saturation (%) *	−0.026	0.974	0.946–1.003	0.075
Albumin (mg/dL) *	−0.550	0.577	0.487–0.684	<0.001
Full functional capacity	0.379	1.460	1.148–1.857	0.002
Full mental status	0.629	1.876	1.416–2.485	<0.001
Solid–organ transplantation	0.601	1.823	1.114–2.983	0.017
Peripheral vascular disease	0.538	1.713	1.124–2.613	0.012
Atrial fibrillation	0.233	1.262	0.981–1.623	0.071
Nasogastric tube	0.810	2.249	1.464–3.454	0.000
Central venous catheter	0.771	2.161	1.251–3.734	0.006
Urinary catheter	0.439	1.551	1.158–2.078	0.003

* Continuous variable: OR per one unit.

**Table 3:** Patients divided into 4 groups with increasing probabilities of antibiotic treatment in the derivation cohort and the two validation ones.

Cohort	*p*-Value *	No. of Patients Treated with Antibiotics/Total (%)
Beilinson	≤0.04	50/1540 (3.2%)
	0.04–0.06	58/1390 (4.2%)
	0.06–0.1	108/1355 (8.0%)
	≥0.1	238/1307 (18.2%)
Hasharon	≤0.04	19/1115 (1.7%)
	0.04–0.06	21/830 (2.5%)
	0.06–0.1	38/590 (6.4%)
	≥0.1	66/526 (12.5%)
Rambam	≤0.04	14/549 (2.6%)
	0.04–0.06	48/1392 (3.4%)
	0.06–0.1	84/1374 (6.1%)
	≥0.1	244/1179 (20.7%)

* Probability of antibiotic treatment as calculated by the regression model.

**Table 4:** Characteristics of patients in the validation cohorts.

	Validation Cohort Hasharon (N = 3061)			Validation Cohort Rambam (N = 4494)		
Variable	Antibiotic Treatment (NO) N = 2917	Antibiotic Treatment (YES) N = 144	*p*-Value	Antibiotic Treatment (NO) N = 4104	Antibiotic Treatment (YES) N = 390	*p*-Value
Hospitalization, days	3 (2–4)	8 (5–15)	<0.001	4 (3–6)	10 (6–16)	<0.001
Gender, female	1457 (49.9%)	86 (59.7%)	0.022	1859 (45.3%)	191 (49%)	0.164
Prophylactic antibiotic treatment	54 (1.9%)	4 (2.8%)	0.426	71 (1.7%)	13 (3.3%)	0.025
Heart rate	79 (68–92)	85 (72–96)	0.008	80 (70–94)	85 (72–96)	0.001
Diastolic blood pressure (mmHg)	73 (63–83)	69 (58–80)	0.001	77 (69–85)	75 (66–81)	<0.001
O2 saturation (%)	98 (97–100)	97 (95–100)	<0.001	97 (95–99)	96 (93–98)	<0.001
Albumin mg/dL	4.2 (3.9–4.5)	3.8 (3.4–4.2)	<0.001	3.7 (3.4–4.0)	3.4 (3.1–3.8)	<0.001
Full functional capacity	1961 (67.2%)	53 (36.8%)	<0.001	2748 (67%)	154 (39.5%)	<0.001
Full mental status	2644 (90.6%)	105 (72.9%)	<0.001	3924 (95.6%)	319 (81.8%)	<0.001
Solid organ transplantation	29 (1%)	2 (1.4%)	0.644	45 (1.1%)	6 (1.5%)	0.431
Peripheral vascular disease	108 (3.7%)	9 (6.3%)	0.12	141 (3.4%)	27 (6.9%)	0.001
Atrial fibrillation	489 (16.8%)	28 (19.4%)	0.4	750 (18.3%)	115 (29.5%)	<0.001
Nasogastric tube	30 (1%)	10 (6.9%)	<0.001	24 (0.6%)	14 (3.6%)	<0.001
Central venous catheter	9 (0.3%)	4 (2.8%)	<0.001	31 (0.8%0	34 (8.7%)	<0.001
Urinary catheter	170 (5.8%)	25 (17.4%)	<0.001	290 (7.1%)	168 (43.1%)	<0.001

Continuous variables are described as median (25–75 percentiles); discrete variables are described as number (%).

## Data Availability

All data are available in the paper.

## Supplementary Materials

Patient flow diagram; Performance metrics. The following supporting information can be downloaded at: www.mdpi.com/article/10.3390/antibiotics11060813/s1.
