# Supplementary material for: Predicting In-Hospital Antibiotic Use in the Medical Department: Derivation and Validation Study
Source: Antibiotics (Basel). 2022 Jun 16;11(6):813. doi: 10.3390/antibiotics11060813 (PMC9219723; doi:10.3390/antibiotics11060813)
Supplement: Supplementary file 1 [file antibiotics-11-00813-s001.zip › antibiotics-1702321-supplementary.pdf]

<u>Patient flow diagram</u>

Derivation cohort – Beilinson Hospital

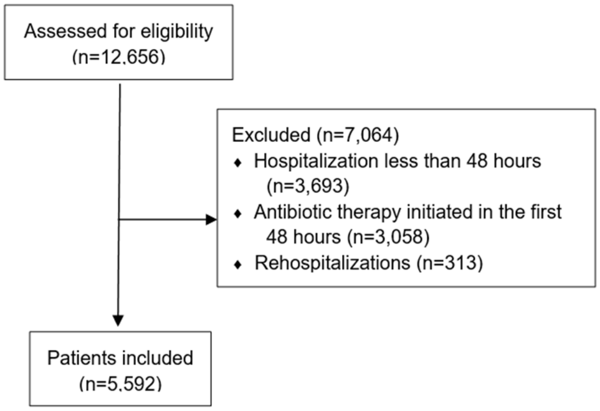

Validation cohort – Hasharon Hospital

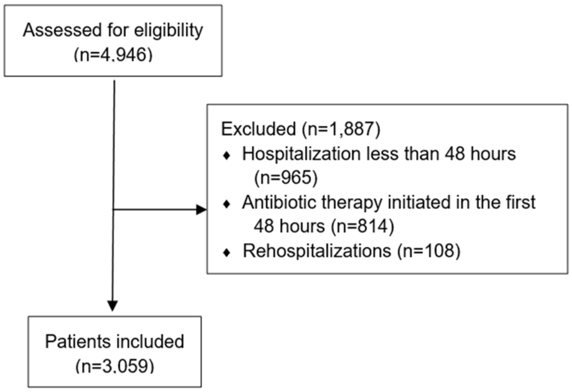

Validation cohort – Rambam Health Care Campus

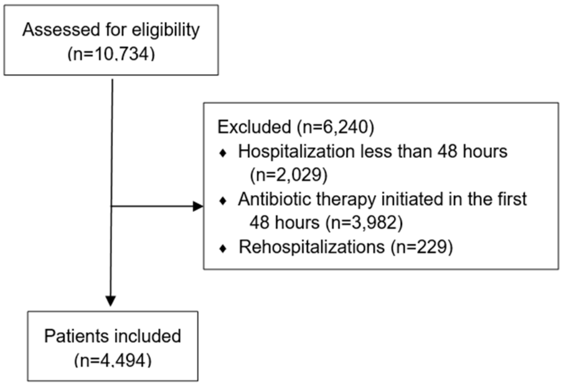

Performance Metrics

**Medical Center**	**Probability Thresholds**	**Sensitivity**	**Specificity**	**Positive Predictive Value**	**Negative Predictive Value**	**Accuracy**
**Beilinson**	0.04	0.89	0.29	0.1	0.97	0.34
	0.06	0.76	0.55	0.13	0.96	0.56
	0.085*	0.63	0.72	0.17	0.95	0.72
	0.1	0.52	0.8	0.18	0.95	0.77
**Hasharon**	0.04	0.87	0.37	0.06	0.98	0.4
	0.06	0.72	0.65	0.09	0.98	0.65
	0.085*	0.49	0.79	0.10	0.97	0.78
	0.1	0.45	0.84	0.12	0.97	0.82
**Rambam Health Care Campus**	0.04	0.96	0.13	0.09	0.97	0.20
	0.06	0.84	0.45	0.13	0.97	0.49
	0.085*	0.69	0.69	0.17	0.96	0.69
	0.1	0.62	0.77	0.20	0.95	0.76

* Youden's index
